# Diversity Patterns of Protists Are Highly Affected by Methods Disentangling Biological Variants: A Case Study in Oligotrich (s.l.) Ciliates

**DOI:** 10.3390/microorganisms10050913

**Published:** 2022-04-27

**Authors:** Jiahui Xu, Jianlin Han, Hua Su, Changyu Zhu, Zijing Quan, Lei Wu, Zhenzhen Yi

**Affiliations:** 1 Guangzhou Key Laboratory of Subtropical Biodiversity and Biomonitoring, Guangdong Provincial Key Laboratory for Healthy and Safe Aquaculture, School of Life Science, South China Normal University, Guangzhou 510631, China; xujiahui@m.scnu.edu.cn (J.X.); 18613650037@163.com (J.H.); sh15603069440@163.com (H.S.); zhuchangyu@m.scnu.edu.cn (C.Z.); jenchuen@126.com (Z.Q.); aleiwoo@163.com (L.W.); 2 Institute of Evolution & Marine Biodiversity, College of Fisheries, Ocean University of China, Qingdao 266003, China

**Keywords:** protist, diversity, environmental sequences, salinity, ecological transition

## Abstract

Protists are a dominant group in marine microplankton communities and play important roles in energy flux and nutrient cycling in marine ecosystems. Environmental sequences produced by high-throughput sequencing (HTS) methods are increasingly used for inferring the diversity and distribution patterns of protists. However, studies testing whether methods disentangling biological variants affect the diversity and distribution patterns of protists using field samples are insufficient. Oligotrich (s.l.) ciliates are one group of the abundant and dominant planktonic protists in coastal waters and open oceans. Using oligotrich (s.l.) ciliates in field samples as an example, the present study indicates that DADA2 performs better than SWARM, UNOISE, UPARSE, and UCLUST for inferring diversity patterns of oligotrich (s.l.) ciliates in the Pearl River Estuary and surrounding regions. UPARSE and UNOISE might underestimate species richness. SWARM might not be suitable for the resolution of alpha diversity owing to its rigorous clustering and sensitivity to sequence variations. UCLUST with 99% clustering threshold overestimates species richness, and the beta diversity pattern inferred by DADA2 is more reasonable than that of the other methods. Additionally, salinity is shown to be one of the key factors responsible for variations in the community distribution of ciliates, but infrequent marine–freshwater transitions occurred during evolutionary terms of this group.

## 1. Introduction

Protists, single-celled eukaryotes, are widely distributed in soil, marine environments, and freshwater worldwide and play key roles in energy flux as well as trophic interactions and nutrient cycling [1,2,3]. They are a dominant group in marine microzooplankton communities and act as primary producers, predators, decomposers, and/or parasites in marine ecosystems [2,4,5]. However, protists with low abundance in a region are easily ignored in observation in vivo due to their small size and difficulty in identification and cultivation [6,7]. Foissner [8] assumed that more than a half of the diversity of many protist groups has not been revealed. Consequently, insufficient sampling of protists in studies using isolated cells might halt revealing their accurate patterns of diversity, phylogeny, and transition. Fortunately, rare and cryptic protists could be revealed by high-throughput sequencing (HTS) with large data output, which provides us a chance to investigate more accurate diversity and distribution patterns of protists [9,10,11,12,13].

Considering that both real biological variants (inter-/intra-specific variants) and spurious sequences introduced by sequencing are included in HTS datasets, a method that disentangles biological variation is one of the main factors affecting the accuracy of downstream analyses [14,15,16,17]. Nowadays, two sequence grouping approaches are popular for disentangling biological variations [14]. One is the construction of operational taxonomic units (OTUs) with different quality filtering and clustering algorithms, e.g., UPARSE [16], UNOISE [18], UCLUST [19], and SWARM [20]; different clustering thresholds of sequences have been reported to define OTUs of protists (e.g., 95% in [21]; 97% in [22]; 98% in [23]; 99% in [24]). Another is correcting amplicon errors by generating Amplicon Sequence Variants (ASVs) at single nucleotide resolution, e.g., DADA2 [25]. To our knowledge, many references focusing on protist diversity based on HTS data are published every year, but there are only a few studies that compare the effects of different HTS data processing methods on analyses [17,26,27]. Some studies suggested that DADA2-derived ASVs could more accurately reproduce a known alpha diversity than SWARM-derived OTUs for ciliate species [27], and USEARCH and QIIME strongly affected the number of predicted OTUs but not the biogeographical patterns of protists [26]. Additionally, the numbers of OTUs varied with the change in clustering thresholds in prokaryotes and protists [28]. Consequently, protist diversity inferred from HTS data is highly affected not only by sequence grouping approaches but also by clustering thresholds producing OTUs. However, some questions are still unclear. For instance, testing whether ASVs and/or OTUs affect beta diversity patterns of protists in real case scenarios using field samples [27].

Oligotrich (s.l.) ciliates are a major group of the abundant and dominant planktonic protist communities in coastal waters and open oceans [1,29,30,31,32,33]. Salinity gradients of the Pearl River Estuary (PRE) and surrounding coasts range from 0.1‰ to 32.0‰ [34]. In the present study, we will explore whether methods disentangling biological variants will affect the diversity patterns of protists by using oligotrich (s.l.) ciliates collected from this region as an example. Our aim is to provide suggestions for data analyses in future studies. Moreover, we investigate the ecological transitions of oligotrich (s.l.) ciliates using both released sequences of identified species in GenBank and environmental sequences from this region since expanded taxa sampling has been proven to be beneficial for classifying transition patterns of ciliates [35].

## 2. Materials and Methods

### 2.1. Environmental Sequences of Oligotrich (s.l.) Ciliates

**Figure 1:**
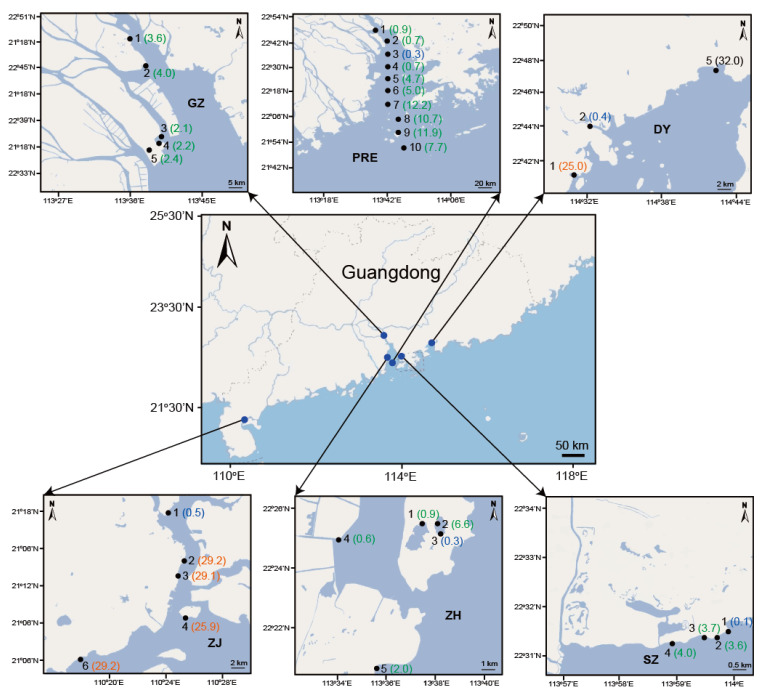
The location of sampling regions. Sampling areas and their abbreviations are as follows: Daya Bay (DY1, DY2, DY5), Pearl River Estuary (PRE1–PRE10), Guangzhou (GZ1–GZ5), Shenzhen (SZ1–SZ4), Zhuhai (ZH1–ZH5), and Zhanjiang (ZJ1–ZJ4, ZJ6). The numbers shown in parentheses represent the salinity of the sampling sites (unit: ‰), and salinity values of freshwater, low-brackish, high-brackish, and marine areas are colored in blue, green, orange, and black, respectively. Adapted with permission from [34] published by John Wiley and Sons, 2022.

Oligotrich (s.l.) ciliates consist of two subclasses, Oligotrichia and Choreotrichia [36]. The environmental SSU rDNA V4 region sequences were downloaded from GenBank under the accession number PRJNA646537. The geographic locations of sampling sites and the salinity of each site in this study are shown in Figure 1. In summary, PRE1–PRE11 are sampling sites along the flow direction of the Pearl River Estuary (PRE), and sampling sites DY1–DY5, GZ1–GZ5, SZ1–SZ6, ZH1–ZH5, and ZJ1–ZJ6 are in the sampling areas Daya Bay (DY), Guangzhou (GZ), Shenzhen (SZ), Zhuhai (ZH), and Zhanjiang (ZJ), respectively. Among these sampling areas, SZ and DY are located on the eastern side of PRE, ZH and ZJ are on the western side of PRE, and GZ is in the upper reaches of PRE. The hydrological and nutritional conditions from the western and eastern sides of PRE are different, and PRE provides a complex ecosystem with various salinity gradients from the upper to lower reaches [34]. According to the Venice Salinity Classification System (Source: Limnology and Oceanography, 1958), the water salinity is classified as freshwater (≤0.5‰, low-brackish (0.5–18.0‰), high-brackish (18.0–30.0‰), and marine (30.0–40.0‰).

To test whether the methods of disentangling biological variants affect diversity patterns, all downloaded data were processed using five frequently used methods: UPARSE and UNOISE in USEARCH v11.0 [16,18], UCLUST in QIIME v1.9.1 [15,19], SWARM v3 [20], and DADA2 in QIIME2 v2019.10 [14,25]. In total, six oligotrich (s.l.) datasets, viz., UPARSE–97, UNOISE–100, UCLUST–97, UCLUST–99, SWARM–100, and DADA2–100, were produced from environmental sequences as follows. For datasets UPARSE–97, UNOISE–100, SWARM–100, UCLUST–97, and UCLUST–99, which produce OTUs, the same quality filtering protocol was performed in order to only compare differences among clustering algorithms. Briefly, the paired-end reads were merged with FLASH [37] and filtered with the following settings: sequences of length <200 or >500, average quality <20, ambiguous bases >0, or homopolymer length >6 were removed using QIIME v1.8.0 [15]. Dereplication and the discarding of singletons were performed by using USEARCH v11.0 [16]. Sequences of UPARSE–97 were clustered into OTUs at the default 97% similarity. Sequences of UNOISE–100 were denoised into zero-radius operational taxonomic units (ZOTUs), which are valid OTUs superior to conventional 97% OTUs, using unoise3 with default parameters [18]. Sequences of SWARM–100 were clustered using SWARM v3 with d = 1 [20] and subsequently subjected to chimera detection using VSEARCH v2.15.0 [38]. And 97% and 99% similarity thresholds were set for the UCLUST algorithm as an example to check whether diversity patterns varied with changes in clustering thresholds, considering that both of them have been reported to define OTUs of protists [22,24]. For DADA2–100, the demultiplexed reads with QIIME2 v2019.10 [25] were filtered and denoised using DADA2 [14] with “––p–trunc–len–f” and “––p–trunc–len–r” parameters set to 240 and 210, respectively. The sequences of each ASV with one nucleotide difference were produced. For all datasets, the Silva 138 SSURef database (https://doi.org/10.5281/ZENODO.3891931, accessed on 17 May 2021) was utilized to annotate taxonomic information for each representative sequence. After that, only sequences annotated as oligotrich (s.l.) ciliates (similarity > 90% as in [26]) were kept in each dataset for downstream analyses. We assumed that these sequences might reflect the abundance and community diversity of oligotrich (s.l.) ciliates even though these sequences are not biologically realistic. DY3, DY4, PRE11, SZ5, SZ6, and ZJ5 were deleted from subsequent analyses because fewer than five oligotrich (s.l.) sequences were detected in these sampling sites by all methods.

In order to explore the diversity patterns of oligotrich (s.l.) ciliates among different datasets, a Bray–Curtis distance matrix was used to quantify the community dissimilarity. Hierarchical clustering and Principal Co-ordinates Analysis (PCoA) were performed in the R platform [39] using the packages “ape” [40] and “vegan” [41]. The sequences of OTUs/ASVs from different analytical methods were log (x + 1) transformed to improve normality and homoscedasticity before downstream analysis. Hierarchical clustering was performed using the “ward.D2” algorithm of the “hclust” function. PCoA was performed based on the Bray–Curtis dissimilarity, and the “stat_ellipse” (level = 0.95) command was used to add ellipses by group except DY (too few points to calculate an ellipse) to visualization results from PCoA. The differences between predefined groups based on sampling areas were statistically tested by permutational multivariate analysis of variance (ADONIS) [42] using 1000 permutations.

### 2.2. Sequence Alignment and Phylogenetic Analyses

All available SSU rDNA sequences (>1000 bp, 191 in total) of identified oligotrich (s.l.) species as of August 2021, as well as six sequences of the subclass Hypotrichia used as the outgroup, were downloaded from GenBank (accession numbers in Supplementary Table S1). The dataset DADA2–100 performed better for inferring diversity patterns of oligotrich (s.l.) ciliates in the Pearl River Estuary and surrounding regions than other five datasets due to more reliable alpha diversity and beta diversity (detailed information in discussion). Hence, the dataset containing 197 identified sequences listed in Supplementary Table S1 and 103 representative sequences from dataset DADA2–100 were aligned using the GUIDANCE2 server [43]. The resulting alignment was manually checked in SeaView v4 [44] for trimming two ends and ambiguous sites.

Maximum likelihood (ML) analyses were carried out using RAxML-HPC2 on XSEDE on CIPRES Science Gateway (http://www.phylo.org/sub_sections/portal, accessed on 30 August 2021) [45] using the GTRGAMMA model, and support for the best-scoring ML tree was assessed by 1000 bootstrap replicates. Bayesian inference (BI) analysis was also performed on CIPRES Science Gateway using MrBayes on XSEDE v3.2.7a, using the GTR+I+G model, which was selected by jModeltest v2.1.10 [46]. Markov chain Monte Carlo simulations were run for 10,000,000 generations with four chains. Trees were sampled every 100 generations, and the first 25% of trees were discarded as burn-in. The 50% majority rule consensus tree was used to calculate the posterior probabilities (PP) for each node. Trees were viewed and edited with FigTree v. 1.4.4 [47]. Finally, Mesquite v3.6 [48] was used to infer the most parsimonious pattern of marine–freshwater transitions of oligotrich (s.l.) ciliates using the ML tree above.

## 3. Results

### 3.1. Diversity Patterns of Oligotrich (s.l.) Ciliates

Since the total number of oligotrich (s.l.) OTUs/ASVs contained in each dataset varied greatly from 63 (UPARSE–97) to 248 (UCLUST–99) (Table 1), the ratio of oligotrich (s.l.) OTUs/ASVs number in each sampling area to the total number of oligotrich (s.l.) OTUs/ASVs within each dataset was also compared (Table 2). The proportions of oligotrich (s.l.) OTUs/ASVs in each sampling area/site in UPARSE–97 and UNOISE–100 were generally higher than those in the other four datasets (Table 2 and Supplementary Table S2). Among the six sampling areas, the largest proportion of oligotrich (s.l.) OTUs/ASVs was detected in PRE, with largest in UPARSE–97 (71.43%) and lowest in DADA2–100 (45.63%). The lowest proportion of oligotrich (s.l.) OTUs/ASVs was detected in DY based on DADA2–100 (5.83%), UCLUST–97 (13.46%), and UCLUST–99 (6.85%) (Table 2). However, the lowest one was detected in SZ based on SWARM–100 (10.95%), UNOISE–100 (13.48%), and UPARSE–97 (15.87%) (Table 2).

**Figure 2:**
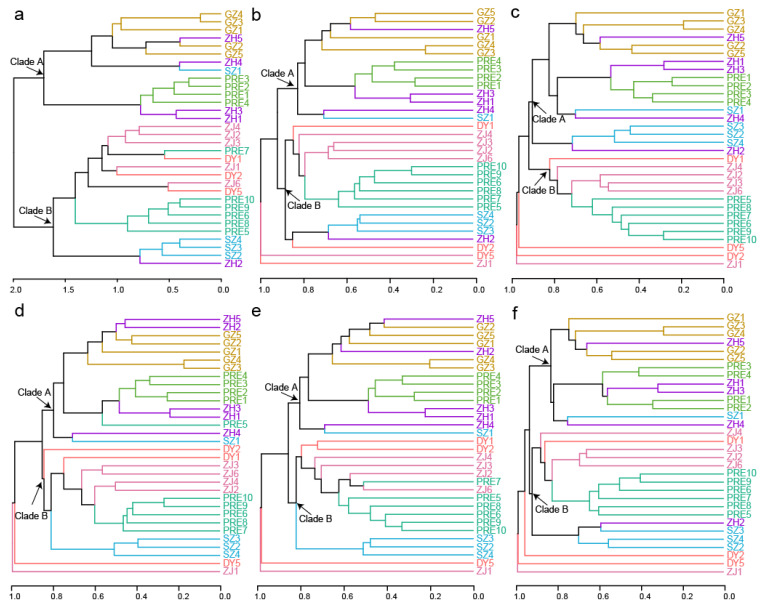
Hierarchical clustering analysis of oligotrich (s.l.) community of 32 samples from six datasets. (**a**) DADA2–100; (**b**) SWARM–100; (**c**) UNOISE–100; (**d**) UPARSE–97; (**e**) UCLUST–97; (**f**) UCLUST–99. Samples from the same sampling area are indicated by the same color except for samples from PRE. Samples named PRE1–4 and PRE5–10 are indicated by different colors.

The hierarchical clustering analyses show that oligotrich (s.l.) samples grouped into two major clades in DADA2–100 (Figure 2a). Generally, Clade A consisted of most samples from sampling areas ZH and GZ, as well as samples of PRE1–PRE4. Clade B contained most samples from sampling areas ZJ, SZ, and DY, as well as samples of PRE5–PRE10. Different from the general clustering pattern in DADA2–100 (Figure 2a), samples ZJ1 and DY5 were far from Clade A and Clade B in SWARM–100, UPARSE–97, and UCLUST–97 (Figure 2b,d,e), and samples ZJ1, DY2, and DY5 were far from Clade A and Clade B in UNOISE–100 and UCLUST–99 (Figure 2c,f). Additionally, sample PRE5 fell into Clade A instead of Clade B in UPARSE–97 (Figure 2d). Notably, community structures of ZJ samples were more similar to each other than to PRE samples in DADA2–100 (Figure 2a). By contrast, some ZJ samples clustered with PRE samples first and then with other ZJ samples in all other five datasets (Figure 2b–f).

**Figure 3:**
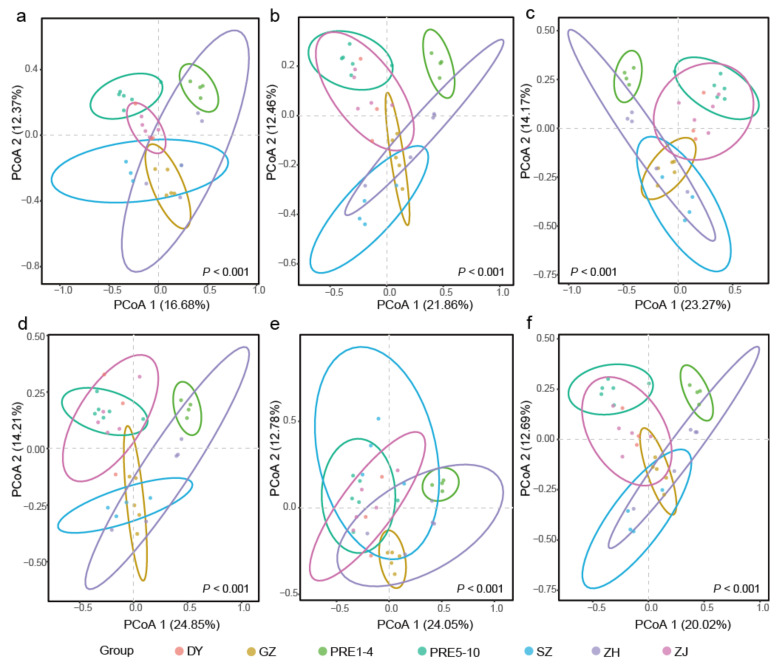
The distributions of oligotrich (s.l.) communities from six datasets ((**a**). DADA2–100; (**b**). SWARM–100; (**c**). UNOISE–100; (**d**). UPARSE–97; (**e**). UCLUST–97; (**f**). UCLUST–99) based on Principal Co-ordinates Analysis (PCoA). Samples from the same sampling area are indicated by the same color except for samples from PRE. Samples named PRE1–4 and PRE5–10 are indicated by different colors. The ellipses represent 95% confidence intervals except for DY (too few samples to calculate an ellipse). *P* represents global significance among oligotrich (s.l.) communities from different sampling areas based on ADONIS. *p* values < 0.05 indicate significant differences among community structures.

PCoA results showed that oligotrich (s.l.) samples were basically grouped depending on the six sampling areas (DY, GZ, PRE, SZ, ZH, ZJ), and PRE was divided into two groups (PRE1–PRE4, PRE5–PRE10) (Figure 3). ADONIS results supported the significant (*p* < 0.001) differentiations of community structures among these seven groups based on all six datasets (Figure 3). Consistent with the hierarchical clustering results (Figure 2), samples of PRE1–PRE4 were clearly separated from those of PRE5–PRE10, ZJ, SZ, and DY in all six datasets (Figure 3). However, for DADA2–100, samples of PRE1–PRE4, ZH, and GZ tended to group together and were separated from other sampling sites (Figure 3a).

### 3.2. Phylogeny and Transition Patterns of Oligotrich (s.l.) Ciliates

**Figure 4:**
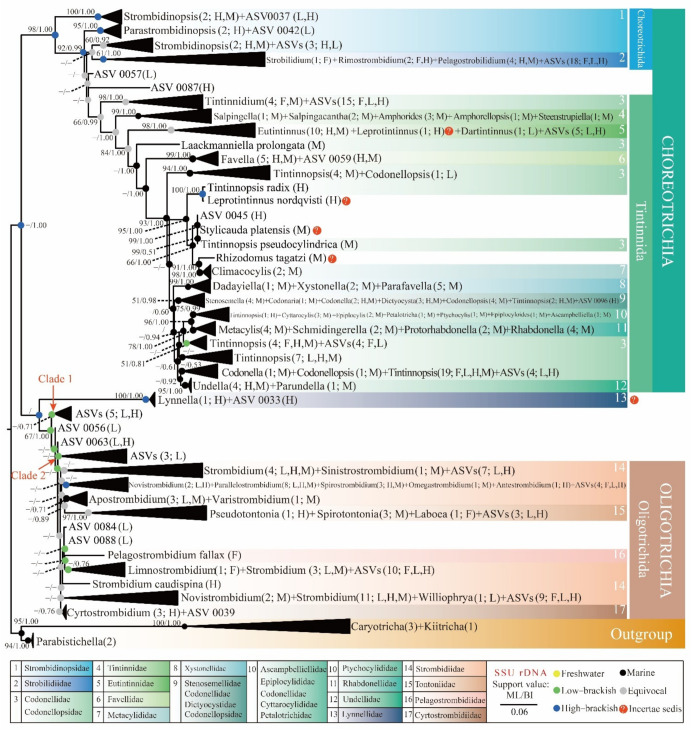
Maximum likelihood (ML) tree based on SSU rDNA of 103 environmental sequences (ASVs in DADA2–100) and 197 sequences of identified species from GenBank (accession numbers in Supplementary Table S1). The tree shows the topology and transition pattern; support values (ML/BI) > 50%/0.50 are labeled. The number of collapsed sequences and ecological characteristics are shown in parentheses. F, freshwater; L, low-brackish; H, high-brackish; M, marine.

To investigate the ecological transition patterns of oligotrich (s.l.) ciliates, a phylogenetic tree including 197 sequences from identified species (Supplementary Table S1) and 103 ASVs from dataset DADA2–100 was constructed (Figure 4), as DADA2–100 yielded more reliable diversity patterns of oligotrich (s.l.) ciliates in the Pearl River Estuary and surrounding regions than the other five datasets. Monophyly of the subclass Choreotrichia was supported by high support (98% ML, 1.00 BI), but that of subclass Oligotrichia was not. In the phylogenetic trees, crown clades usually contained identified oligotrich (s.l.) species and ASVs from various habitats. Though most ASV groups had identified species, some ASVs formed isolated clades (Clade 1, Clade 2 in Figure 4) without identified species. This indicates that oligotrich (s.l.) diversity in low-brackish habitats might have been underestimated in previous morphological investigations. Transition pattern analyses showed a high-brackish ancestor for oligotrich (s.l.) ciliates (Figure 4). The subclass Choreotrichia appeared to have evolved from the high-brackish ancestor with high support values (98% ML, 1.00 BI), with some transitions to freshwater, low-brackish, and marine areas and even back to high-brackish for some species (e.g., *Tintinnopsis radix* and *Leprotintinnus nordqvisti*) (Figure 4). By contrast, the subclass Oligotrichia seemed to be derived from an equivocal ancestor with poor support values (ML < 50%, BI < 0.50), with some transition to freshwater, low brackish, high-brackish, and marine areas and possible additional transition back to low-brackish areas for some species (e.g., *Parallelostrombidium paraellipticum* and *P. obesum*) (Figure 4).

## 4. Discussion

### 4.1. Methods Disentangling Biological Variants Highly Affect Diversity Patterns of Oligotrich (s.l.) Ciliates

The alpha and beta diversities of six oligotrich (s.l.) datasets were compared in order to check which method performed best for inferring diversity patterns of oligotrich (s.l.) ciliates in the Pearl River Estuary and surrounding regions. The detailed discussion is as following:

Among six oligotrich (s.l.) datasets generated by different algorithms and thresholds (viz. DADA2–100, SWARM–100, UNOISE–100, UPARSE–97, UCLUST–97, UCLUST–99), the actual number of oligotrich (s.l.) OTU/ASV as well as ratio of the oligotrich (s.l.) OTUs/ASVs number in each sample area/site were different (Table 1 and Table 2, Supplementary Table S2). This is consistent with previous investigations that the alpha diversity of protists is highly variable depending on sequence grouping approaches, as well as software and clustering thresholds producing OTUs [26,27,28,49]. Recent investigations revealed that compared with OTUs, ASVs could more accurately reproduce a known alpha diversity from mock communities of various groups, e.g., bacteria [14,50,51], fungi [50], ciliated protists [27]. In this study, the number of oligotrich (s.l.) OTUs/ASVs inferred by UCLUST–99 (248) is unreasonable (Table 1), because only 288 morphological oligotrich (s.l.) ciliate species were reported in the South China Sea from 1991 to 2018 [52]. The numbers of oligotrich (s.l.) OTUs/ASVs inferred by UPARSE–97 (63) and UNOISE–100 (89) were much lower than those in the other three datasets (103–137) (Table 1). One possible explanation is that UPARSE and UNOISE could not detect fine-scale or low-abundance biological variations [14,18]. Previous studies showed that SWARM was not suitable for the resolution of genetic diversity and alpha diversity in the samples with high intraspecific sequence variations due to its rigorous clustering and sensitivity to sequence variations [20,27,53]. Only alpha diversity patterns revealed by DADA2–100 and UCLUST–97 seemed be reliable. Interestingly, numbers of ASVs/OTUs are comparable between DADA2–100 (103) and UCLUST–97 (104), but the ratio of oligotrich (s.l.) OTU/ASV numbers in each sampling area to the total number of oligotrich (s.l.) OTUs/ASVs within each dataset was rather different between these two datasets (Table 1 and Table 2). This indicates that beta diversity patterns should be compared between these two datasets in order to check which one is better.

In this study, a general beta diversity pattern of oligotrich (s.l.) ciliates was revealed in the six oligotrich (s.l.) datasets, but methods disentangling biological variants also had an impact on the beta diversity patterns (Figure 2 and Figure 3). Clearly, community variations were observed between PRE1–PRE4 and PRE5–PRE10 in DADA2–100, SWARM–100, UNOISE–100, UCLUST–97 and UCLUST–99, and PRE5 groups with PRE1–PRE4 instead of PRE6–PRE10 in UPARSE–97. This might be explained by the sharp increase in salinity between sampling sites PRE1–PRE4 (0.3–0.9‰) and PRE5–PRE10 (4.7–12.2‰). It is possible that the mixture of freshwater and seawater formed a low-salinity front between sampling sites PRE4 and PRE5 [54]. Numerous studies have proven that salinity appeared to be the factor that correlated best with distributions of phytoplankton and bacterioplankton in estuaries (e.g., [55,56,57,58,59]) and hence community compositions of oligotrich (s.l.) ciliates with phytoplankton and bacterioplankton as food also changed greatly between PRE1–PRE4 and PRE5–PRE10. Similar to PRE1–PRE4, sampling sites GZ1–GZ5 are in the upper estuary of the Pearl River Estuary and are highly influenced by large freshwater discharge from the Pearl River. Theoretically, community structures of oligotrich (s.l.) ciliates in the upper Pearl River Estuary with lower salinity and higher nutrient content should be much different from those in the lower Pearl River Estuary. In the present study, the GZ samples were grouped with PRE1–PRE4 in all six datasets (Figure 2 and Figure 3). Hierarchical clustering analyses of DADA2–100, SWARM–100, UPARSE–97, UCLUST–97, and UCLUST–99 showed that most ZH samples grouped with PRE1–PRE4 in Clade A, and most SZ samples fell into Clade B including PRE5–PRE10 (Figure 2a,b,d,f), although sampling areas ZH and SZ are located at a similar latitude of PRE5. Different community structures of oligotrich (s.l.) ciliates in ZH and SZ samples might be due to following reason. The surface flow velocity on the western side (ZH) is usually greater than that on the eastern side (SZ) of the Pearl River Estuary [60]. This indicates that community structures of oligotrich (s.l.) ciliates in ZH might be more highly influenced by river runoff than in SZ. Thus, cluster patterns of most SZ and ZH samples falling into Clade A in UNOISE–100 (Figure 2c) were less reliable than in the other five datasets (Figure 2a,b,d,f). Sampling areas ZJ and DY are located in the nearshore area of the South China Sea (surrounding regions of the Pearl River Estuary), and most samples from these sampling areas clustered with PRE5–PRE10 in all six datasets (Figure 2 and Figure 3). Possibly, salinity also plays an important role in this cluster pattern, since salinity values in sampling areas ZJ, DY and sampling sites PRE5–PRE10 were generally higher than those in other areas (Figure 1). Notably, both the geographical locations and salinity of sampling sites ZJ2–ZJ4 and ZJ6 were very similar to each other (Figure 1), but their close relationships based on hierarchical clustering analysis were only as obvious in DADA2–100 (Figure 2a). Additionally, three samples collected from DY area fell into a subclade of Clade B in DADA2–100, while DY5 always formed a separate basal clade in the other five datasets (Figure 2b–f). Hence, DADA2–100 showed the most reliable clustering patterns for ZJ and DY samples, although a general beta diversity pattern of oligotrich (s.l.) ciliates was revealed in the six oligotrich (s.l.) datasets. All these results reveal that the beta diversity pattern inferred by DADA2–100 was more reasonable in real case scenarios using field samples of oligotrich (s.l.) ciliates.

In sum, among the six datasets compared in this study, DADA2–100 performed best for inferring the diversity pattern of oligotrich (s.l.) ciliates in the Pearl River Estuary and surrounding regions. As described in the Introduction, this is the first study to test whether ASVs and/or OTUs affect the beta diversity patterns of protists in real case scenarios. In future, more studies using filed samples are expected to test whether ASVs perform best for inferring diversity pattern of protists in various regions.

### 4.2. Community Distribution and Ecological Transitions of Oligotrich (s.l.) Ciliates in Environments with Various Salinity Gradients

As revealed in various groups of archaea, bacteria, and protists (e.g., [34,55,57,58,61]), the present study also revealed that salinity gradients play a vital role in structuring patterns for the community distribution of oligotrich (s.l.) ciliates in the Pearl River Estuary and surrounding regions that encompass an entire freshwater–marine salinity gradient. First, communities of oligotrich (s.l.) ciliates in sampling sites PRE1–PRE10 along the flow direction of the Pearl River Estuary were divided into two distinct groups PRE1–PRE4 (Clade A) and PRE5–PRE10 (Clade B) (Figure 2a), which is consistent with the sharp change in salinity gradients between sampling sites PRE1–PRE4 (0.3–0.9‰) and PRE5–PRE10 (4.7–12.2‰). Additionally, all samples from high-brackish and marine habitats fell into Clade B, though these samples were collected from two sampling areas (ZJ and DY) of around 500 km. It is believed that both the physicochemical barrier of salinity gradients and the presence of locally adapted taxa limit the colonization success of microbes in different habitats with salinity gradients [62]. Within Clade B, several samples (DY2, ZJ1, PRE5–PRE10, SZ2–SZ4) were from freshwater and low-brackish habitats, which indicates that in addition to salinity, other environmental factors and geographical distance also shape the community distribution of oligotrich (s.l.) ciliates. This was also revealed in various prokaryotic and eukaryotic microbial groups in estuaries (e.g., [34,52,63]).

As mentioned above, salinity gradients have been proven to be important physicochemical factor in structuring community distribution and limiting transitions of microbes including protists. Ancestors of different ciliate groups seem to originate in various habitats. Oligotrich (s.l.) and hypotrich (present study, [35]) ciliates are ancestrally high-brackish, and peritrich and colpodean ciliates have freshwater/terrestrial ancestors [64,65]. However, species in various habitats are reported for each of these ciliate groups. That is because infrequent marine–freshwater transitions always occurred during their evolutionary terms (present study; [35,64,65,66]) due to the ability of microbes to rapidly adapt and highly colonize to new environments [62,67]. Environmental SSU rDNA V4 region sequences of the Pearl River Estuary and surrounding coasts cover oligotrich (s.l.) ciliates in various salinity gradients (0.1–32.0‰) [34], providing us a good chance to classify ecological transition patterns of this taxonomy group with expanded taxa sampling [35]. Although the short fragments of SSU rDNA have limited phylogenetic signals [64], previous studies have proven that the addition of SSU rDNA V4 region sequences produced from amplicons could improve the ecological transition patterns of ciliates by broad taxa sampling in various habits [35,64]. In our phylogenetic trees (Figure 4), some ASVs formed separate clades (Clade 1, Clade2) and might be new taxa. This is consistent with a previous report that the oligotrich (s.l.) morphospecies diversity was underestimated [68]. Additionally, these ASVs representing new taxa were from low-brackish habitats (Figure 4). By contrast, among all SSU rDNA sequences of identified oligotrich (s.l.) species deposited in GenBank, only 4% (8 out of 191) and 8% (15 out of 191) were from freshwater and low-brackish habitats (Supplementary Table S1). In future, more detailed transitions and evolutionary patterns of oligotrich (s.l.) ciliates are expected be outlined with broader sampling, especially freshwater and low-brackish habitats.

## 5. Conclusions

This study investigates whether methods disentangling biological variants will affect the diversity patterns of protists by using oligotrich (s.l.) ciliates in field samples as a case. Our work demonstrates that DADA2 performed better than SWARM, UNOISE, UPARSE, and UCLUST for inferring diversity patterns of oligotrich (s.l.) ciliates. In addition, salinity was shown to be one of the key factors responsible for variations in the community distribution of ciliates but infrequent marine–freshwater transitions occurred during the evolutionary terms of this group.

## Figures and Tables

**Table 1:** The number of total and oligotrich (s.l.) OTUs/ASVs in six datasets.

	DADA2–100	SWARM–100	UNOISE–100	UPARSE–97	UCLUST–97	UCLUST–99
Number of total OTUs/ASVs	3890	6656	2915	3095	7613	19,993
Number of oligotrich (s.l.) OTUs/ASVs	103	137	89	63	104	248

**Table 2:** Number and proportion of oligotrich (s.l.) OTUs/ASVs in each sampling area from the six datasets. The proportions in parentheses indicate the ratio of the oligotrich (s.l.) OTU/ASV number in each sampling area to the total number of oligotrich (s.l.) OTUs/ASVs within each dataset.

Sampling Areas	DADA2–100	SWARM–100	UNOISE–100	UPARSE–97	UCLUST–97	UCLUST–99
DY	6 (5.83%)	17 (12.41%)	14 (15.73%)	15 (23.81%)	14 (13.46%)	17 (6.85%)
GZ	31 (30.10%)	54 (39.42%)	49 (55.06%)	31 (49.21%)	39 (37.50%)	88 (35.48%)
PRE	47 (45.63%)	76 (55.47%)	58 (65.17%)	45 (71.43%)	67 (64.42%)	137 (55.24%)
SZ	8 (7.77%)	15 (10.95%)	12 (13.48%)	10 (15.87%)	16 (15.38%)	30 (12.10%)
ZH	23 (22.33%)	40 (29.20%)	40 (44.94%)	28 (44.44%)	27 (25.96%)	51 (20.56%)
ZJ	32 (31.07%)	63 (45.99%)	46 (51.69%)	32 (50.79%)	45 (43.27%)	110 (44.35%)

## Data Availability

The data generated and/or analyzed in this study are available from the corresponding author upon reasonable request.

## Supplementary Materials

The following supporting information can be downloaded at: https://www.mdpi.com/article/10.3390/microorganisms10050913/s1, Table S1: Detailed information of identified oligotrich (s.l.) ciliate species from GenBank. All sequences are SSU rDNA sequences. Ecological habitat is classified as F = freshwater (≤0.5‰), L = low-brackish (0.5–18‰), H =high-brackish (18–30‰), and M = marine (30–40‰). References [66,69,70,71,72,73,74,75,76,77,78,79,80,81,82,83,84,85,86,87,88,89,90,91,92,93,94,95,96,97,98,99,100,101,102,103,104,105,106,107,108,109,110,111,112,113,114,115,116,117,118,119,120,121,122,123,124,125,126,127,128,129,130,131,132,133,134,135,136,137,138,139,140,141,142,143,144,145,146,147,148,149,150,151,152] are cited in the Supplementary Materials. Table S2: Number and proportion of oligotrich (s.l.) OTUs/ASVs in each sampling site from the six datasets.
