# Supplementary material for: Diversity Patterns of Protists Are Highly Affected by Methods Disentangling Biological Variants: A Case Study in Oligotrich (s.l.) Ciliates
Source: Microorganisms. 2022 Apr 27;10(5):913. doi: 10.3390/microorganisms10050913 (PMC9147439; doi:10.3390/microorganisms10050913)
Supplement: Supplementary file 1 [file microorganisms-10-00913-s001.zip › microorganisms-1637444-supplementary.pdf]

Table S1. Detailed information of identified oligotrich (s.l.) species from GenBank. All sequences are SSU rDNA sequences. Ecological habitat is classified as F = freshwater (≤ 0.5‰), L = low−brackish (0.5–18‰), H = high−brackish (18–30‰), and M = marine (30–40‰).

Species	Accession Number	Habitat Type	Data Source	Species	Accession Number	Habitat Type	Data Source
Spirotontonia grandis	KU525755	M	[1]	*Parallelostrombidium obesum*	FJ422991	L	[49]
Tintinnopsis fimbriata	AY143560	M	[2]	*Strombidium basimorphum*	FJ480419	L	[50]
Strombidium triquetrum	KJ609052	L	[3]	*Williophrya maedai*	FJ876966	L	[50]
Apostrombidium pseudokielum	MH688479	M	[4]	*Sinistrostrombidium cupiformum*	JX310366	M	[51]
Strombidium biarmatum	AY541684	M	[4]	*Strombidium tropicum*	KJ609050	M	[51]
Pelagostrobilidium neptuni	AY541683	M	[4]	*Strombidinopsis batos*	FJ881862	M	[52]
Parabistichella variabilis	JN008943	F	[4]	*Caryotricha minuta*	EU275202	M	[53]
Stenosemella pacifica	JN831791	M	[5]	*Novistrombidium testaceum*	AJ488910	M	[54]
Codonaria cistellula	JQ408202	M	[6]	*Strombidium inclinatum*	AJ488911	M	[54]
Tintinnopsis lacustris	JQ408161	F	[6]	*Eutintinnus lususundae*	AB640633	M	[55]
Codonellopsis orthoceras	JQ408180	M	[6]	*Favella markusovszkyi*	JN871725	M	[55]
Dictyocysta reticulata	EU399532	M	[6]	*Xystonella longicauda*	KT792933	M	[55]
Rhabdonella elegans	JQ408175	M	[6]	*Caryotricha marina*	MG603637	M	[56]
Tintinnidium fluviatile	JQ408163	F	[6]	*Metacylis angulata*	KY290322	M	[57]
Undella marsupialis	JQ408214	M	[6]	*Stylicauda platensis*	JN831832	M	[57]
Favella adriatica	JQ408215	M	[6]	*Laboea strobila*	AF399151	F	[58]
Tintinnopsis rara	JQ408199	M	[6]	*Codonella amphorella*	AB640629	M	[59]
Tintinnopsis orientalis	MK036422	H	[7]	*Tintinnopsis baltica*	JN831806	M	[59]
Tintinnopsis tentaculata	MK036423	H	[7]	*Cyttarocylis cassis*	JQ408203	M	[59]
Tintinnopsis hemispiralis	MT435073	H	[8]	*Cyttarocylis eucecryphalus*	JQ408186	M	[59]
Tintinnopsis kiaochowensis	MT435074	H	[8]	*Epiplocylis acuminata*	JQ715615	M	[59]
Limnostrombidium viride	KU525754	F	[9]	*Epiplocylis undella*	KY290319	M	[59]
Strombidium purpureum	U97112	L	[10]	*Protorhabdonella simplex*	KY290323	M	[59]

Tintinnopsis beroidea	EF123709	M	[11]
Tintinnopsis lobiancoi	AB640667	M	[11]
Dictyocysta lepida	KT792929	M	[11]
Climacocylis scalaroides	KY290330	M	[11]
Protorhabdonella curta	JX101863	M	[11]
Amphorellopsis acuta	JX101848	M	[11]
Dadayiella ganymedes	JX101852	M	[11]
Eutintinnus apertus	JQ408195	M	[11]
Eutintinnus fraknoi	JQ408159	M	[11]
Eutintinnus medius	KT792925	M	[11]
Eutintinnus tenuis	JN871721	M	[11]
Eutintinnus tubulosus	JX101855	M	[11]
Steenstrupiella steenstrupii	KT792924	M	[11]
Tintinnopsis ventricosoides	KU715776	H	[12]
Pelagostrobilidium liui	JQ781699	M	[13]
Eutintinnus pectinis	JN871720	H	[14]
Tintinnopsis acuminata	JN831839	H	[15]
Tintinnidium balechi	JN831797	M	[16]
Leprotintinnus pellucidus	AB640641	H	[16]
Tintinnopsis buetschlii	JN831810	M	[17]
Eutintinnus perminutus	KT792926	M	[18]
Tintinnopsis tubulosoides	AF399108	M	[19]
Cyttarocylis acutiformis	KY290316	M	[20]
Pseudotontonia simplicidens	FJ422993	H	[21]
Stenosemella nivalis	AB640648	M	[22]
Tintinnopsis everta	MG461220	H	[23]
Eutintinnus stramentus	JX101859	M	[59]
Salpingella acuminata	EU399536	M	[59]
Undella claparedei	JQ408190	M	[59]
Undella subcaudata	KT792931	M	[59]
Parundella aculeata	KY290327	M	[59]
Petalotricha ampulla	KY290317	M	[60]
Ptychocylis minor	KY290321	M	[60]
Salpingacantha undata	KY290325	M	[60]
Salpingacantha unguiculata	KY290326	M	[60]
Xystonella acus	KY290329	M	[60]
Strombidium apolatum	DQ662848	H	[61]
Tintinnopsis kiangsuensis	JN831849	M	[62]
Tintinnopsis parva	JN831824	M	[62]
Tintinnopsis pseudocylindrica	JN831855	M	[62]
Tintinnopsis turbinata	JN831846	M	[62]
Tintinnopsis urnula	JN831852	M	[62]
Tintinnopsis lata	KM982810	H	[63]
Tintinnopsis levigata	KM982811	H	[63]
Dartintinnus alderae	MF039886	L	[64]
Strombidium chlorophilum	KM084726	M	[65]
Strombidium oculatum	KM084727	M	[65]
Strombidium pseudostylifer	KM084728	H	[65]
Strombidium stylifer	JX012185	H	[65]
Apostrombidium parakielum	JX025560	M	[66]
Novistrombidium apsheronicum	FJ876958	M	[66]
Pelagostrombidium fallax	MK110374	F	[67]

Codonella aspera	JQ408179	H	[24]	*Cyrtostrombidium paraboreale*	MH688481	H	[68]
Dictyocysta elegans	KY290318	H	[24]	*Apostrombidium orientale*	MH688480	L	[68]
Strombidinopsis jeokjo	AJ628250	H	[25]	*Parallelostrombidium dragescoi*	MF445658	H	[69]
Caryotricha rariseta	FJ876978	H	[26]	*Parallelostrombidium jankowskii*	MF445659	H	[69]
Parafavella greenlandica	MH673399	M	[27]	*Parallelostrombidium kahli*	MF445656	M	[69]
Parafavella jorgenseni	MH673382	M	[27]	*Parallelostrombidium paraellipticum*	MF445657	L	[69]
Parafavella subrotundata	MH673386	M	[27]	*Omegastrombidium elegans*	KU525750	M	[70]
Schmidingerella arcuata	JQ837815	M	[28]	*Strombidium sulcatum*	FJ377546	M	[70]
Tintinnopsis lohmanni	FJ196076	F	[29]	*Strombidium capitatum*	KP260510	M	[71]
Favella azorica	AB640634	M	[30]	*Strombidium caudispina*	KP260513	H	[71]
Tintinnopsis mortenseni	AB640672	M	[31]	*Strombidium cuneiforme*	KP260512	L	[71]
Tintinnopsis tubulosa	AB640683	M	[31]	*Strombidium guangdongense*	KJ609049	H	[71]
Tintinnopsis tenuis	AB640679	M	[31]	*Strombidium paracapitatum*	KP260511	L	[71]
Parabistichella multilineae	MK265247	M	[32]	*Strombidinopsis sinicum*	KR263893	H	[71]
Parastrombidinopsis shimi	AJ786648	H	[33]	*Spirostrombidium apourceolare*	KU525746	H	[72]
Laackmanniella prolongata	KY980346	M	[34]	*Strobilidium caudatum*	AY143573	F	[73]
Favella ehrenbergii	GU574770	M	[35]	*Rhabdonella hebe*	AY143566	M	[73]
Strombidinopsis acuminata	FJ790207	M	[36]	*Spirotontonia taiwanica*	FJ715634	M	[74]
Ascampbelliella acuta	KY290315	M	[37]	*Cyrtostrombidium longisomum*	KJ534582	H	[75]
Tintinnopsis elongata	AB640656	M	[37]	*Cyrtostrombidium paralongisomum*	KJ609053	H	[75]
Tintinnopsis uruguayensis	JN831838	M	[37]	*Parastrombidinopsis minima*	DQ393786	H	[76]
Codonellopsis americana	AY143571	M	[37]	*Tintinnopsis gracilis*	MT764266	M	[77]
Codonellopsis morchella	AB640630	M	[37]	*Tintinnopsis karajacensis*	MT757137	H	[77]
Codonellopsis nipponica	KY980414	M	[37]	*Rimostrombidium lacustris*	DQ986131	F	[78]
Stenosemella steini	KT792927	M	[37]	*Spirostrombidium schizostomum*	KM222098	M	[79]
Climacocylis scalaria	JQ408213	M	[37]	*Parallelostrombidium paralatum*	KU525748	H	[80]

Metacylis joergenseni	JQ408183	M	[37]	*Varistrombidium kielum*	KU525749	M	[81]
Rhabdonella poculum	JX101864	M	[37]	*Epiplocyloides ralumensis*	JX101854	M	[82]
Rhabdonella spiralis	KT792932	M	[37]	*Metacylis pithos*	JX101862	M	[82]
Amphorides minor	KY290324	M	[37]	*Amphorides amphora*	JX101849	M	[82]
Undella hyalina	JQ408207	H	[37]	*Amphorides quadrilineata*	JX101850	M	[82]
Tintinnopsis rapa	JN831834	M	[37]	*Tintinnopsis brasiliensis*	KU715768	M	[83]
Codonella apicata	EU399531	M	[38]	*Tintinnopsis cylindrica*	KU715769	M	[83]
Strombidium paracalkinsi	KJ737432	H	[39]	*Tintinnopsis fistularis*	KU715770	M	[83]
Spirotontonia turbinata	FJ422994	M	[40]	*Tintinnopsis parvula*	KU715771	M	[83]
Metacylis tropica	KP883283	M	[41]	*Tintinnopsis radix*	KU715772	H	[83]
Strombidium pseudorapulum	MT274320	H	[42]	*Stenosemella ventricosa*	KU715764	M	[83]
Parafavella gigantea	MH673409	M	[43]	*Rhizodomus tagatzi*	KU715762	M	[83]
Parafavella parumdentata	KY290328	M	[43]	*Schmidingerella quequenensis*	KU715765	M	[83]
Kiitricha marina	AY896768	M	[44]	*Eutintinnus cf. apertus*	KU715759	H	[83]
Novistrombidium orientale	FJ422988	L	[45]	*Tintinnidium mucicola*	KU715767	M	[83]
Novistrombidium sinicum	FJ422989	H	[45]	*Tintinnidium cf. primitivum*	KU715766	M	[83]
Parallelostrombidium conicum	JN712657	H	[46]	*Leprotintinnus nordqvisti*	KU715761	H	[83]
Spirostrombidium subtropicum	JN712658	H	[46]	*Favella panamensis*	KU715760	H	[83]
Lynnella semiglobulosa	FJ876965	H	[47]	*Codonellopsis mobilis*	MK799838	M	[84]
Pelagostrobilidium minutum	FJ876959	M	[48]	*Tintinnopsis chinglanensis*	MK799839	H	[85]
Pelagostrobilidium paraepacrum	FJ876963	H	[48]	*Tintinnopsis nana*	JN831821	L	[85]
Rimostrombidium veniliae	FJ876964	H	[48]	*Tintinnopsis tocantinensis*	JN831837	L	[85]
Antestrombidium agathae	JX310365	H	[49]	*Codonellopsis ostenfeldi*	AB640631	L	[85]
Parallelostrombidium ellipticum	KJ704987	L	[49]				

Table S2. Number and proportion of oligotrich (s.l.) OTUs/ASVs in each sampling site from the six datasets.

Sampling Sites	DADA2–100	SWARM–100	UNOISE–100	UPARSE–97	UCLUST–97	UCLUST–99
DY1	3 (2.86%)	8 (5.84%)	6 (6.74%)	8 (12.70%)	8 (7.69%)	8 (3.23%)
DY2	2 (1.90%)	6 (4.38%)	4 (4.49%)	5 (7.94%)	5 (4.81%)	6 (2.42%)
DY5	1 (0.95%)	5 (3.65%)	4 (4.49%)	5 (7.94%)	5 (4.81%)	5 (2.02%)
GZ1	7 (6.67%)	16 (11.68%)	20 (22.47%)	13 (20.63%)	15 (14.42%)	23 (9.27%)
GZ2	8 (7.62%)	14 (10.22%)	14 (15.73%)	11 (17.46%)	12 (11.54%)	20 (8.06%)
GZ3	16 (15.24%)	33 (24.09%)	27 (30.34%)	21 (33.33%)	23 (22.12%)	46 (18.55%)
GZ4	14 (13.33%)	31 (22.63%)	25 (28.09%)	21 (33.33%)	22 (21.15%)	51 (20.56%)
GZ5	13 (12.38%)	20 (14.6%)	22 (24.72%)	19 (30.16%)	17 (16.35%)	35 (14.11%)
PR1	20 (19.05%)	39 (28.47%)	28 (31.46%)	26 (41.27%)	35 (33.65%)	67 (27.02%)
PR2	9 (8.57%)	33 (24.09%)	23 (25.84%)	22 (34.92%)	30 (28.85%)	55 (22.18%)
PR3	13 (12.38%)	25 (18.25%)	21 (23.60%)	19 (30.16%)	19 (18.27%)	32 (12.9%)
PR4	12 (11.43%)	19 (13.87%)	18 (20.22%)	16 (25.40%)	19 (18.27%)	22 (8.87%)
PR5	7 (6.67%)	28 (20.44%)	25 (28.09%)	20 (31.75%)	26 (25.00%)	48 (19.35%)
PR6	11 (10.48%)	23 (16.79%)	20 (22.47%)	14 (22.22%)	20 (19.23%)	44 (17.74%)
PR7	10 (9.52%)	14 (10.22%)	13 (14.61%)	9 (14.29%)	10 (9.62%)	18 (7.26%)
PR8	5 (4.76%)	16 (11.68%)	13 (14.61%)	12 (19.05%)	15 (14.42%)	27 (10.89%)
PR9	5 (4.76%)	25 (18.25%)	22 (24.72%)	21 (33.33%)	23 (22.12%)	42 (16.94%)
PR10	11 (10.48%)	18 (13.14%)	15 (16.85%)	13 (20.63%)	18 (17.31%)	32 (12.9%)
SZ1	3 (2.86%)	6 (4.38%)	6 (6.74%)	6 (9.52%)	7 (6.73%)	9 (3.63%)
SZ2	6 (5.71%)	11 (8.03%)	9 (10.11%)	7 (11.11%)	11 (10.58%)	23 (9.27%)
SZ3	4 (3.81%)	7 (5.11%)	7 (7.87%)	7 (11.11%)	7 (6.73%)	7 (2.82%)
SZ4	3 (2.86%)	5 (3.65%)	4 (4.49%)	4 (6.35%)	6 (5.77%)	10 (4.03%)
ZH1	11 (10.48%)	25 (18.25%)	24 (26.97%)	21 (33.33%)	19 (18.27%)	29 (11.69%)
ZH2	5 (4.76%)	8 (5.84%)	8 (8.99%)	8 (12.70%)	5 (4.81%)	10 (4.03%)
ZH3	15 (14.29%)	30 (21.9%)	29 (32.58%)	22 (34.92%)	23 (22.12%)	39 (15.73%)
ZH4	2 (1.90%)	8 (5.84%)	9 (10.11%)	7 (11.11%)	7 (6.73%)	9 (3.63%)
ZH5	8 (7.62%)	15 (10.95%)	16 (17.98%)	11 (17.46%)	11 (10.58%)	16 (6.45%)
ZJ1	1 (0.95%)	3 (2.19%)	3 (3.37%)	2 (3.17%)	1 (0.96%)	3 (1.21%)
ZJ2	14 (13.33%)	30 (21.9%)	23 (25.84%)	22 (34.92%)	21 (20.19%)	38 (15.32%)
ZJ3	5 (4.76%)	22 (16.06%)	18 (20.22%)	18 (28.57%)	15 (14.42%)	22 (8.87%)
ZJ4	17 (16.19%)	41 (29.93%)	37 (41.57%)	22 (34.92%)	34 (32.69%)	88 (35.48%)
ZJ6	3 (2.86%)	13 (9.49%)	12 (13.48%)	12 (19.05%)	13 (12.50%)	15 (6.05%)

References

1. Agatha, S. New observations on the tontoniid ciliate *Spirotontonia grandis* (Suzuki and Han, 2000) Agatha, 2004 (Ciliophora, Oligotrichida, Tontoniidae); comparison with the similar *Laboea strobila*. *Eur J Protistol* **2004,** *40*, 295–301. https://doi.org/10.1016/j.ejop.2004.06.001
2. Agatha, S. Redescription of the tintinnid ciliate *Tintinnopsis fimbriata* Meunier, 1919 (Spirotricha, Choreotrichida) from coastal waters of northern Germany. *Denisia* **2008,** *23*, 261–272.
3. Agatha, S.; Riedel-Lorjé, J.C. Morphology, infraciliature, and ecology of Halteriids and Strombidiids (Ciliophora, Oligotrichea) from coastal brackish water basins. *Arch Protistenkd* **1997,** *148*, 445–459. https://doi.org/10.1016/S0003-9365(97)80021-8
4. Agatha, S.; Strüder-Kypke, M.C.; Beran, A.; Lynn, D.H. *Pelagostrobilidium neptuni* (Montagnes and Taylor, 1994) and *Strombidium biarmatum* nov. spec. (Ciliophora, Oligotrichea): phylogenetic position inferred from morphology, ontogenesis, and gene sequence data. *Eur J Protistol* **2005,** *41*, 65–83. https://doi.org/10.1016/j.ejop.2004.09.005
5. Agatha, S.; Tsai, S.F. Redescription of the tintinnid *Stenosemella pacifica* Kofoid and Campbell, 1929 (Ciliophora, Spirotricha) based on live observation, protargol impregnation, and scanning electron microscopy. *J Eukaryot Microbiol* **2008,** *55*, 75–85. https://doi.org/10.1111/j.1550-7408.2008.00309.x
6. Bachy, C.; Gómez, F.; López-García, P.; Dolan, J.R.; Moreira, D. Molecular phylogeny of tintinnid ciliates (Tintinnida, Ciliophora). *Protist* **2012,** *163*, 873–887. https://doi.org/10.1016/j.protis.2012.01.001
7. Bai, Y.; Wang, R.; Liu, W.; Warren, A.; Zhao, Y.; Hu, X. Redescriptions of three tintinnine ciliates (Ciliophora: Tintinnina) from coastal waters in China based on lorica features, cell morphology, and rDNA sequence data. *Eur J Protistol* **2020,** *72*, 125659. https://doi.org/10.1016/j.ejop.2019.125659
8. Bai, Y.; Wang, R.; Song, W.; Li, L.; Santoferrara, L.F.; Hu, X. Three redescriptions in Tintinnopsis (Protista: Ciliophora: Tintinnina) from coastal waters of China, with cytology and phylogenetic analyses based on ribosomal RNA genes. *BMC Microbiol* **2020,** *20*, 1–19. https://doi.org/10.1186/s12866-020-02057-2
9. Bardele, C.F.; Stockmann, N.; Agatha, S. Some ultrastructural features of the planktonic freshwater ciliate *Limnostrombidium viride* (Alveolata, Ciliophora, Oligotrichida) and improved diagnoses of oligotrich taxa. *Acta Protozool* **2019,** *57*, 169–193. https://doi.org/10.4467/16890027AP.18.014.10090
10. Bernard, C.; Fenchel, T. Chemosensory behaviour of *Strombidium purpureum*, an anaerobic oligotrich with endosymbiotic purple non-sulphur bacteria. *J Eukaryot Microbiol* **1994,** *41*, 391–396. https://doi.org/10.1111/j.1550-7408.1994.tb06095.x
11. Cariou, J.B.; Dolan, J.; Dallot, S. A preliminary study of tintinnid diversity in the NW Mediterranean Sea. *J Plankton Res* **1999,** *21*, 1065–1075. https://doi.org/10.1093/plankt/21.6.1065
12. Cedrola, F.; Senra, M.V.X.; D'Agosto, M.; Dias, R.J.P. Phylogenetic analyses support validity of genus *Eodinium* (Ciliophora, Entodiniomorphida, Ophryoscolecidae). *J Eukaryot Microbiol* **2017,** *64*, 242–247. https://doi.org/10.1111/jeu.12355
13. Chen, P.C.; Chiang, K.P.; Tsai, S.F. *Pelagostrobilidium liui* n. sp. (Ciliophora, Choreotrichida) from the coastal waters of northeastern Taiwan and an improved description of *Pelagostrobilidium minutum* Liu et al., 2012. *J Eukaryot Microbiol* **2017,** *64*, 579–587. https://doi.org/10.1111/jeu.12392
14. Coats, D.W. *Duboscquella cachoni* n. sp. a parasitic dinoflagellate lethal to its tintinnine host *Eutintinnus pectinis*. *J Eukaryot Microbiol* **1988,** *35*, 607–617. https://doi.org/10.1111/j.1550-7408.1988.tb04159.x
15. Coats, D.W.; Heinbokel, J.A. Study of reproduction and other life cycle phenomena in planktonic protists using an acridine orange fluorescence technique. *Mar Biol* **1982,** *67*, 71–79. https://doi.org/10.1007/BF00397096
16. de Cao, M.B.; Beigt, D.; Piccolo, C. Temporal variability of diversity and biomass of tintinnids (Ciliophora) in a southwestern Atlantic temperate estuary. *J Plankton Res* **2005,** *27*, 1103–1111. https://doi.org/10.1093/plankt/fbi077
17. de Cao, M.B. Abundance and species composition of Tintinnina (Ciliophora) in Bahía Blanca estuary, Argentina. *Estuar Coast Shelf Sci* **1992,** *34*, 295–303. https://doi.org/10.1016/S0272-7714(05)80085-X
18. Santoferrara, L.F.; Grattepanche, J.D.; Katz, L.A.; McManus, G.B. Patterns and processes in microbial biogeography: do molecules and morphologies give the same answers? *ISME J* **2016,** *10*, 1779–1790. https://doi.org/10.1038/ismej.2015.224
19. Feng, M.; Zhang, W.; Wang, W.; Zhang, G.; Xiao, T.; Xu, H. Can tintinnids be used for discriminating water quality status in marine ecosystems? *Mar Pollut Bull* **2015,** *101*, 549–555. https://doi.org/10.1016/j.marpolbul.2015.10.059
20. Feng, M.; Zhang, W.; Yu, Y.; Xiao, T.; Sun, J. Horizontal distribution of tintinnids in the western

South China Sea during summer 2007 (in Chinese). *Trop Oceanogr* **2013,** *32*, 86–92. https://doi.org/10.3969/j.issn.1009-5470.2013.03.013
21. Gao, S.; Gong, J.; Lynn, D.; Lin, X.; Song, W. An updated phylogeny of oligotrich and choreotrich ciliates (Protozoa, Ciliophora, Spirotrichea) with representative taxa collected from Chinese coastal waters. *Syst Biodivers* **2009,** *7*, 235–242. https://doi.org/10.1017/S1477200009002989
22. Graziano, C. On the ecology of tintinnids (Ciliophora: Oligotrichida) in the North Irish Sea. *Estuar Coast Shelf Sci* **1989,** *29*, 233–245. https://doi.org/10.1016/0272-7714(89)90055-3
23. Gruber, M.S.; Strüder-Kypke, M.; Agatha, S. Redescription of *Tintinnopsis everta* Kofoid and Campbell 1929 (Alveolata, Ciliophora, Tintinnina) based on taxonomic and genetic analyses—discovery of a new complex ciliary pattern. *J Eukaryot Microbiol* **2018,** *65*, 484–504. https://doi.org/10.1111/jeu.12496
24. Urrutxurtu, I. Seasonal succession of tintinnids in the Nervión River estuary, Basque Country, Spain. *J Plankton Res* **2004,** *26*, 307–314. https://doi.org/10.1093/plankt/fbh034
25. Jeong, H.J.; Kim, J.S.; Kim, S.; Song, J; Lee, I.; Lee, G.H. *Strombidinopsis jeokjo* n. sp. (Ciliophora: Choreotrichida) from the coastal waters off western Korea: morphology and small subunit ribosomal DNA gene sequence. *J Eukaryot Microbiol* **2004,** *51*, 451–455. https://doi.org/10.1111/j.1550-7408.2004.tb00393.x
26. Jiang, J.; Xing, Y.; Miao, M.; Shao, C.; Warren, A.; Song, W. Two new marine ciliates, *Caryotricha rariseta* n. sp. and *Discocephalus pararotatorius* n. sp.(Ciliophora, Spirotrichea), with phylogenetic analyses inferred from the small subunit rRNA gene sequences. *J Eukaryot Microbiol* **2013,** *60*, 388–398. https://doi.org/10.1111/jeu.12046
27. Jung, J.H.; Moon, J.H.; Park, K.M.; Kim, S.; Dolan, J.R.; Yang, E.J. Novel insights into the genetic diversity of *Parafavella* based on mitochondrial CO1 sequences. *Zool Scr* **2018,** *47*, 743–755. https://doi.org/10.1111/zsc.12312
28. Jung, J.H.; Choi, J.M.; Coats, D.W.; Kim, Y.O. *Euduboscquella costata* n. sp.(Dinoflagellata, Syndinea), an intracellular parasite of the ciliate *Schmidingerella arcuata*: morphology, molecular phylogeny, life cycle, prevalence, and infection intensity. *J Eukaryot Microbiol* **2016,** *63*, 3–15. https://doi.org/10.1111/jeu.12231
29. Jyothibabu, R.; Madhu, N.; Jayalakshmi, K.; Balachandran, K.; Shiyas, C.; Martin, G.; Nair, K. Impact of freshwater influx on microzooplankton mediated food web in a tropical estuary (Cochin backwaters–India). *Estuar Coast Shelf Sci* **2006,** *69*, 505–518. https://doi.org/10.1016/j.ecss.2006.05.013
30. Kamiyama, T. Growth and grazing responses of tintinnid ciliates feeding on the toxic dinoflagellate Heterocapsacircularisquama. *Mar Biol* **1997,** *128*, 509–515. https://doi.org/10.1007/s002270050117
31. Kazama, T.; Ishida, S.; Shimano, S.; Urabe, J. Discrepancy between conventional morphological systematics and nuclear phylogeny of tintinnids (Ciliophora: Choreotrichia). *Plankton Benthos Res* **2012,** *7*, 111–125. https://doi.org/10.3800/pbr.7.111
32. Kim, J.H.; Omar, A.; Jung, J.H. Morphology and phylogeny of the soil ciliate *Parabistichella multilineae* sp. nov. (Protozoa: Ciliophora: Hypotricha). *Zool Sci* **2019,** *36*, 242–249. https://doi.org/10.2108/zs180070
33. Kim, J.S.; Jeong, H.J.; Strüder-Kypke, M.C.; Lynn, D.H.; Kim, S.; Kim, J.H.; Lee, S.H. *Parastrombidinopsis shimi* n. gen., n. sp. (Ciliophora: Choreotrichia) from the coastal waters of Korea: morphology and small subunit ribosomal DNA sequence. *J Eukaryot Microbiol* **2005,** *52*, 514–522. https://doi.org/10.1111/j.1550-7408.2005.00062.x
34. Kim, S.Y.; Choi, J.K.; Dolan, J.R.; Shin, H.C.; Lee, S.; Yang, E.J. Morphological and ribosomal DNA-based characterization of six antarctic ciliate morphospecies from the Amundsen Sea with phylogenetic analyses. *J Eukaryot Microbiol* **2013,** *60*, 497–513. https://doi.org/10.1111/jeu.12057
35. Kim, S.Y.; Yang, E.J.; Gong, J.; Choi, J.K. Redescription of *Favella ehrenbergii* (Claparède and Lachmann, 1858) Jörgensen, 1924 (Ciliophora: Choreotrichia), with phylogenetic analyses based on small subunit rRNA gene sequences. *J Eukaryot Microbiol* **2010,** *57*, 460–467. https://doi.org/10.1111/j.1550-7408.2010.00500.x
36. Kim, Y.O.; Kim, S.Y.; Lee, W.J.; Choi, J.K. New observations on the choreotrich ciliate *Strombidinopsis acuminata* Fauré-Fremiet 1924, and comparison with *Strombidinopsis jeokjo* Jeong et al., 2004. *J Eukaryot Microbiol* **2010,** *57*, 48–55. https://doi.org/10.1111/j.1550-7408.2009.00446.x
37. Kim, Y.O.; Shin, K.; Jang, P.G.; Choi, H.W.; Noh, J.H.; Yang, E.J.; Kim, E.; Jeon, D. Tintinnid species as biological indicators for monitoring intrusion of the warm oceanic waters into Korean coastal waters. *Ocean Sci J* **2012,** *47*, 161–172. https://doi.org/10.1007/s12601-012-0016-4
38. Kršinić, F. On vertical distribution of tintinnines (Ciliata, Oligotrichida, Tintinnina) in the open waters of the South Adriatic. *Deep-Sea Res Part B Oceanogr Lit Rev* **1982,** *29*, 83–90. https://doi.org/10.1016/0198-0254(82)90374-0

39. Lee, E.S.; Kim, Y.O.; Agatha, S.; Jung, J.H.; Xu, D.; Shin, M.K. Revision of *Strombidium paracalkinsi* (Ciliophora: Oligotrichea: Oligotrichia), with comparison of Strombidiids bearing thigmotactic membranelles. *J Eukaryot Microbiol* **2015,** *62*, 400–409. https://doi.org/10.1111/jeu.12195
40. Lee, E.S.; Shin, M.K.; Kim, Y.O. Morphological descriptions of four oligotrich ciliates (Ciliophora: Oligotrichia) from southern coast of Korea. *Korean J Syst Zool* **2011,** *27,* 131–141. https://doi.org/10.5635/KJSZ.2011.27.2.131
41. Lee, K.W.; Choi, Y.U. Population growth of a tropical tintinnid, Metacylis tropica on different temperature, salinity and diet. *J Korea Acad Industr Coop Soc* **2016,** *17,* 322–328. https://doi.org/10.5762/KAIS.2016.17.9.322
42. Li, F.; Huang, Y.; Yu, Y.; Liu, W.; Lin, X. Taxonomy and phylogeny of a new marine planktonic ciliate, *Strombidium pseudorapulum* sp. n. (Protozoa, Ciliophora, Oligotrichia) (in Chinese). *J Ocean Univ* **2020,** *19,* 954–960. https://doi.org/10.1007/s11802-020-4402-8
43. Li, H.; Xu, Z.; Zhang, W.; Wang, S.; Zhang, G.; Xiao, T. Boreal tintinnid assemblage in the Northwest Pacific and its connection with the Japan Sea in summer 2014. *PloS ONE* **2016,** *11,* e0153379. https://doi.org/10.1371/journal.pone.0153379
44. Li, L.; Shao, C.; Song, W.; Lynn, D.H.; Chen, Z.; Shin, M.K. Does *Kiitricha* (Protista, Ciliophora, Spirotrichea) belong to Euplotida or represent a primordial spirotrichous taxon? With suggestion to establish a new subclass Protohypotrichia. *Int J Syst Evol Microbiol* **2009,** *59,* 439–446. https://doi.org/10.1099/ijs.0.65801-0
45. Liu, W.; Xu, D.; Lin, X.; Li, J.; Gong, J.; Al-Rasheid, K.A.; Song, W. *Novistrombidium sinicum* n. sp. and *Novistrombidium orientale* n. sp.(Protozoa: Ciliophora): two new oligotrich ciliates from a mangrove wetland, South China. *J Eukaryot Microbiol* **2009,** *56,* 459–465. https://doi.org/10.1111/j.1550-7408.2009.00425.x
46. Liu, W.; Yi, Z.; Li, J.; Warren, A.; Al-Farraj, S.A.; Lin, X. Taxonomy, morphology and phylogeny of three new oligotrich ciliates (Protozoa, Ciliophora, Oligotrichia) from southern China. *Int J Syst Evol Microbiol* **2013,** *63,* 4805–4817. https://doi.org/10.1099/ijs.0.052878-0
47. Liu, W.; Yi, Z.; Lin, X.; Al-Rasheid, K.A. Morphologic and molecular data suggest that *Lynnella semiglobulosa* ng, n. sp. represents a new family within the subclass Choreotrichia (Ciliophora, Spirotrichea). *J Eukaryot Microbiol* **2011,** *58,* 43–49. https://doi.org/10.1111/j.1550-7408.2010.00519.x
48. Liu, W.; Yi, Z.; Lin, X.; Warren, A.; Song, W. Phylogeny of three choreotrich genera (Protozoa, Ciliophora, Spirotrichea), with morphological, morphogenetic and molecular investigations on three strobilidiid species. *Zool Scr* **2012,** *41,* 417–434. https://doi.org/10.1080/14772000.2011.605812
49. Liu, W.; Yi, Z.; Lin, X.; Li, J.; Al-Farraj, S.A.; Al-Rasheid, K.A.; Song, W. Morphology and molecular phylogeny of three new oligotrich ciliates (Protozoa, Ciliophora) from the South China Sea. *Zool J Linn Soc* **2015,** *174,* 653–665. https://doi.org/10.1111/zoj.12257
50. Liu, W.; Yi, Z.; Warren, A.; Al-Rasheid, K.A.; Al-Farraj, S.A.; Lin, X.; Song, W. Taxonomy, morphology and molecular systematics of a new oligotrich ciliate, *Williophrya maedai* gen. nov., sp. nov., with redescriptions of *Strombidium basimorphum* and *Pseudotontonia simplicidens* (Protozoa, Ciliophora, Oligotrichia). *Syst Biodivers* **2011,** *9,* 247–258. https://doi.org/10.1080/14772000.2011.605812
51. Liu, W.; Yi, Z.; Xu, D.; Clamp, J.C.; Li, J.; Lin, X.; Song, W. Two new genera of planktonic ciliates and insights into the evolution of the family Strombidiidae (Protista, Ciliophora, Oligotrichia). *PloS ONE* **2015,** *10,* e0131726. https://doi.org/10.1371/journal.pone.0131726
52. Lynn, D.; Montagnes, D.; Dale, T.; Gilron, G.; Strom, S. A reassessment of the genus *Strombidinopsis* (Ciliophora, Choreotrichida) with descriptions of four new planktonic species and remarks in its taxonomy and phylogeny. *J Mar Biol Assoc U K* **1991,** *71,* 597–612. https://doi.org/10.1017/S0025315400053170
53. Miao, M.; Shao, C.; Jiang, J.; Li, L.; Stoeck, T.; Song, W. *Caryotricha minuta* (Xu et al., 2008) nov. comb., a unique marine ciliate (Protista, Ciliophora, Spirotrichea), with phylogenetic analysis of the ambiguous genus *Caryotricha* inferred from the small-subunit rRNA gene sequence. *Int J Syst Evol Microbiol* **2009,** *59,* 430–438. https://doi.org/10.1099/ijs.0.65855-0
54. Modeo, L.; Petroni, G.; Rosati, G.; Montagnes, D.J. A multidisciplinary approach to describe protists: redescriptions of *Novistrombidium testaceum* Anigstein 1914 and *Strombidium inclinatum* Montagnes, Taylor, and Lynn 1990 (Ciliophora, Oligotrichia). *J Eukaryot Microbiol* **2003,** *50,* 175–189. https://doi.org/10.1111/j.1550-7408.2003.tb00114.x
55. Modigh, M.; Castaldo, S.; Saggiomo, M.; Santarpia, I. Distribution of tintinnid species from 42° N to 43° S through the Indian Ocean. *Hydrobiologia* **2003,** *503,* 251–262. https://doi.org/10.1111/j.1550-7408.2003.tb00114.x
56. Park, T.; Yu, Z. Do ruminal ciliates select their preys and prokaryotic symbionts? *Front Microbiol* **2018,** *9,* 1710. https://doi.org/10.3389/fmicb.2018.01710

57. Pierce, R.W.; Turner, J.T. Plankton studies in buzzards Bay, Massachusetts, USA. IV. Tintinnids, 1987 to 1988. *Mar Ecol-Prog Ser* **1994,** *112*, 235–240. https://doi.org/10.3354/meps112235
58. Putt, M. Metabolism of photosynthate in the chloroplast-retaining ciliate *Laboea strobila*. *Mar Ecol-Prog Ser* **1990,** *60*, 271–282. https://doi.org/10.3354/meps060271
59. Sitran, R.; Bergamasco, A.; Decembrini, F.; Guglielmo, L. Temporal succession of tintinnids in the northern Ionian Sea, Central Mediterranean. *J Plankton Res* **2007,** *29*, 495–508. https://doi.org/10.1093/plankt/fbm032
60. Santoferrara, L.F.; Alder, V.V.; McManus, G.B. Phylogeny, classification and diversity of Choreotrichia and Oligotrichia (Ciliophora, Spirotrichea). *Mol Phylogenet Evol* **2017,** *112*, 12–22. https://doi.org/10.1016/j.ympev.2017.03.010
61. Santoferrara, L.F.; McManus, G.B.; Alder, V.A. Phylogeny of the order Tintinnida (Ciliophora, Spirotrichea) inferred from small-and large-subunit rRNA genes. *J Eukaryot Microbiol* **2012,** *59*, 423–426. https://doi.org/10.1111/j.1550-7408.2012.00627.x
62. Santoferrara, L.F.; McManus, G.B.; Alder, V.A. Utility of genetic markers and morphology for species discrimination within the order Tintinnida (Ciliophora, Spirotrichea). *Protist* **2013,** *164*, 24–36. https://doi.org/10.1016/j.protis.2011.12.002
63. Santoferrara, L.F.; Tian, M.; Alder, V.A.; McManus, G.B. Discrimination of closely related species in tintinnid ciliates: new insights on crypticity and polymorphism in the genus *Helicostomella*. *Protist* **2015,** *166*, 78–92. https://doi.org/10.1016/j.protis.2014.11.005
64. Smith, S.A.; Song, W.; Gavrilova, N.A.; Kurilov, A.V.; Liu, W.; McManus, G.B.; Santoferrara, L.F. *Dartintinnus alderae* n. g., n. sp., a brackish water tintinnid (Ciliophora, Spirotrichea) with dual-ended lorica collapsibility. *J Eukaryot Microbiol* **2018,** *65*, 400–411. https://doi.org/10.1111/jeu.12485
65. Song, W.; Li, J.; Liu, W.; Al-Rasheid, K.A.; Hu, X.; Lin, X. Taxonomy and molecular phylogeny of four *Strombidium* species, including description of *S. pseudostylifer* sp. nov. (Ciliophora, Oligotrichia). *Syst Biodivers* **2015,** *13*, 76–92. https://doi.org/10.1080/14772000.2014.970674
66. Song, W.; Li, J.; Liu, W.; Jiang, J.; Al-Rasheid, K.A.; Hu, X. Taxonomy, morphology and molecular systematics of three oligotrich ciliates, including a description of *Apostrombidium parakielum* spec. nov.(Ciliophora, Oligotrichia). *Int J Syst Evol Microbiol* **2013,** *63*, 1179–1191. https://doi.org/10.1099/ijs.0.048314-0
67. Song, W.; Pan, B.; El-Serehy, H.A.; Al-Farraj, S.A.; Liu, W.; Li, L. Morphology and molecular phylogeny of two freshwater oligotrich ciliates (Protozoa, Ciliophora, Oligotrichia), *Pelagostrombidium fallax* (Zacharias, 1895) Krainer, 1991 and *Limnostrombidium viride* (Stein, 1867) Krainer, 1995, with brief notes on stomatogenesis. *J Eukaryot Microbiol* **2020,** *67*, 232–244. https://doi.org/10.1111/jeu.12777
68. Song, W.; Xu, D.; Zhang, Q.; Liu, W.; Warren, A.; Song, W. Taxonomy and phylogeny of two poorly studied genera of marine oligotrich ciliates including descriptions of two new species: *Cyrtostrombidium paraboreale* sp. n. and *Apostrombidium orientale* sp. n.(Ciliophora: Spirotrichea). *Eur J Protistol* **2019,** *70*, 1–16. https://doi.org/10.1016/j.ejop.2019.05.001
69. Song, W.; Wang, L.; Li, L.; Al-Farraj, S.A.; Aleidan, A.; Smith, S.; Hu, X. Morphological characterizations of four species of *Parallelostrombidium* (Ciliophora, Oligotrichia), with a note on the phylogeny of the genus. *J Eukaryot Microbiol* **2018,** *65*, 679–693. https://doi.org/10.1111/jeu.12513
70. Song, W.; Wang, M.; Warren, A. Redescriptions of three marine ciliates, *Strombidium elegans* Florentin, 1901, *Strombidium sulcatum* Claparede & Lachmann, 1859 and *Heterostrombidium paracalkinsi* Lei, Xu & Song, 1999 (Ciliophora, Oligotrichida). *Eur J Protistol* **2000,** *36*, 327–342. https://doi.org/10.1016/S0932-4739(00)80010-3
71. Song, W.; Zhao, X.; Liu, W.; Hu, X.; Al-Farraj, S.A.; Al-Rasheid, K.A.; Song, W.; Warren, A. Biodiversity of oligotrich ciliates in the South China Sea: description of three new *Strombidium* species (Protozoa, Ciliophora, Oligotrichia) with phylogenetic analyses. *Syst Biodivers* **2015,** *13*, 608–623. https://doi.org/10.1080/14772000.2015.1081992
72. Song, Y.; Liu, Y.; Pan, B.; Luo, X.; Song, W.; Warren, A. Morphological studies on four brackish water ciliates of the class Spirotrichea (Protista, Ciliophora). *J Ocean Univ* **2019,** *18*, 663–674. https://doi.org/10.1007/s11802-019-4096-y
73. Strüder-Kypke, M.C.; Lynn, D.H. Sequence analyses of the small subunit rRNA gene confirm the paraphyly of oligotrich ciliates sensu lato and support the monophyly of the subclasses Oligotrichia and Choreotrichia (Ciliophora, Spirotrichea). *J Zool* **2003,** *260*, 87–97. https://doi.org/10.1017/S0952836903003546
74. Tsai, S.F.; Chen, J.Y.; Chiang, K.P. *Spirotontonia taiwanica* n. sp.(Ciliophora: Oligotrichida) from the coastal waters of Northeastern Taiwan: morphology and nuclear small subunit rDNA sequence. *J Eukaryot Microbiol* **2010,** *57*, 429–434. https://doi.org/10.1111/j.1550-7408.2010.00494.x
75. Tsai, S.F.; Chen, W.T.; Chiang, K.P. Phylogenetic position of the genus *Cyrtostrombidium*, with a

description of *Cyrtostrombidium paralongisomum* nov. spec. and a redescription of *Cyrtostrombidium longisomum* Lynn & Gilron, 1993 (Protozoa, Ciliophora) based on live observation, protargol impregnation, and 18S rDNA sequences. *J Eukaryot Microbiol* **2015,** *62*, 239–248. https://doi.org/10.1111/jeu.12173
76. Tsai, S.F.; Xu, D.; Chung, C.C.; Chiang, K.P. *Parastrombidinopsis minima* n. sp. (Ciliophora: Oligotrichia) from the coastal waters of northeastern Taiwan: morphology and small subunit ribosomal DNA sequence. *J Eukaryot Microbiol* **2008,** *55*, 567–573. https://doi.org/10.1111/j.1550-7408.2008.00364.x
77. Wang, R.; Bai, Y.; Hu, T.; Xu, D.; Suzuki, T.; Hu, X. Integrative taxonomy and molecular phylogeny of three poorly known tintinnine ciliates, with the establishment of a new genus (Protista; Ciliophora; Oligotrichea). *BMC Ecol Evol* **2021,** *21*, 1–23. https://doi.org/10.1186/s12862-021-01831-8
78. Weisse, T.; Rammer, S. Pronounced ecophysiological clonal differences of two common freshwater ciliates, *Coleps spetai* (Prostomatida) and *Rimostrombidium lacustris* (Oligotrichida), challenge the morphospecies concept. *J Plankton Res* **2006,** *28*, 55–63. https://doi.org/10.1093/plankt/fbi100
79. Xu, D.; Song, W.; Lin, X.; Warren, A. On two marine oligotrich ciliates, *Spirostrombidium agathae* n. sp. and *S. schizostomum* (Kahl, 1932) n. comb. from China, with a key to the identification of seven well-characterized *Spirostrombidium* spp. (Ciliophora: Oligotrichida). *Acta Protozool* **2006,** *45*, 433–442.
80. Xu, D.; Song, W.; Warren, A. Morphology and infraciliature of two new species of marine oligotrich ciliates (Ciliophora: Oligotrichida) from China. *J Nat Hist* **2006,** *40*, 1287–1299. https://doi.org/10.1080/00222930600913925
81. Xu, D.; Sun, P.; Clamp, J.C.; Ma, H.; Song, W. The establishment of a new oligotrich genus *Varistrombidium* gen. nov. and the morphology and phylogeny of a marine ciliate, *Varistrombidium kielum* (Maeda and Carey, 1985) nov. comb. (Protista, Ciliophora). *Acta Zootaxa Sin* **2011,** *36*, 502–510.
82. Xu, D.; Sun, P.; Warren, A.; Hoon Noh, J.; Lim Choi, D.; Kyoon Shin, M.; Ok Kim, Y. Phylogenetic investigations on ten genera of tintinnid ciliates (Ciliophora: Spirotrichea: Tintinnida), based on small subunit ribosomal RNA gene sequences. *J Eukaryot Microbiol* **2013,** *60*, 192–202. https://doi.org/10.1111/jeu.12023
83. Zhang, Q.; Agatha, S.; Zhang, W.; Dong, J.; Yu, Y.; Jiao, N.; Gong, J. Three rDNA loci-based phylogenies of tintinnid ciliates (Ciliophora, Spirotrichea, Choreotrichida). *J Eukaryot Microbiol* **2017,** *64*, 226–241. https://doi.org/10.1111/jeu.12354
84. Zhang, W.; Sun, J.; Sun, S. Spatial distribution of tintinnidous ciliate *CodoneHopsis mobilis* (Protozoa, Ciliophora) in April, 1999 in Bohai Sea, China. *Marine Sciences* **2004** *28*, 67–69.
85. Zhang, W.; Wang, R. Summertime ciliate and copepod nauplii distributions and micro-zooplankton herbivorous activity in the Laizhou Bay, Bohai Sea, China. *Estuar Coast Shelf Sci* **2000,** *51*, 103–114. https://doi.org/10.1006/ecss.2000.0644
